# The INkWELL Index of Global Innovation

**DOI:** 10.29024/aogh.2363

**Published:** 2018-11-05

**Authors:** Hutan Ashrafian

**Affiliations:** 1 Institute of Global Health Innovation, Imperial College London, GB

## Abstract

The INkWELL index is introduced as a new global innovation index whereby country-level innovation scores can be associated with country-level wellbeing scores, a standard to reflect the ultimate reward of innovation. It is calculated as a composite of the wellbeing score from a nation with its mean national innovation score from the preceding ‘k’ years. The value of k reflects the time-lag from innovation-to-translation into practice that traditional studies have offered a value for 17 years in healthcare. The INkWELL (or INnovation-k-WELLbeing) index can highlight the most innovation-on-wellbeing countries and also offers the assessment of globalization of innovations. As such, this index will offer a meaningful measure of innovation on its ultimate aim of societal wellbeing.

The assessment of country-level innovation through indices such as the Global Innovation Index (GII – https://www.globalinnovationindex.org) offers a useful perception into national education standards, business outcomes, national political effects and infrastructure as a marker with which to guide national policy. There remains however a prominent elephant in the room; when benchmarking a value such as innovation, what is the standard to which it is indexed or normalized?

I suggest a new innovation index whereby country-level innovation scores can be associated with country-level wellbeing scores, the latter being a standard to which I believe is the ultimate prize of innovation.

To calculate this index, the wellbeing score from a nation (www.gallup.com) is multiplied by its mean national innovation score (from the GII) from the preceding ‘k’ years. The value of k reflects the time-lag from innovation-to-translation into practice. For healthcare innovations, k has typically been set at 17 years [1], though for different scientific sectors this can vary considerably. When calculated for 2014, the newly defined INkWELL (or INnovation-k-WELLbeing) index with a k of six years highlights the top innovation-on-wellbeing countries as: Switzerland, Denmark and the Netherlands. Applying such an approach will also allow the assessment of globalization of innovations, as global data can also be appraised in this way. As such, this index will offer a meaningful measure of innovation on its ultimate aim of societal wellbeing.
